# A Manufacturing Technique for Binary Clathrate Hydrates for Cold and Very Cold Neutron Production

**DOI:** 10.3390/ma18020298

**Published:** 2025-01-10

**Authors:** Valentin Czamler, Arnaud Desmedt, Thomas C. Hansen, Richard Wagner, Oliver Zimmer

**Affiliations:** 1 Institut Laue-Langevin (ILL), 71 Avenue des Martyrs, 38042 Grenoble Cedex 9, Francezimmer@ill.fr (O.Z.); 2 École Doctorale de Physique, Université Grenoble Alpes, 38402 Saint Martin d’Hères, France; 3 Institut des Sciences Moléculaires, UMR5255 CNRS—Université de Bordeaux I, 351 Cours de la Libération, 33405 Talence, France; 4 Laboratoire Léon Brillouin, UMR12 CEA-CNRS, Bât. 563 CEA Saclay, 91191 Gif sur Yvette, France

**Keywords:** clathrate hydrates, neutron scattering, neutron diffraction, neutron moderation, neutron sources, very cold neutrons

## Abstract

Intense sources of very cold neutrons (VCNs) would be beneficial for various neutron scattering techniques and low-energy particle physics experiments. Binary clathrate hydrates hosting deuterated tetrahydrofuran (THF-d) and dioxygen show promise as potential moderators for such sources due to a rich spectrum of localized low-energy excitations of the encaged guest molecules. In this article, we present a reliable manufacturing technique for such hydrates. Neutron diffraction data confirm their clathrate structure as type II (CS-II), determine their purity, and cage occupancy. Furthermore, we present data on the thermal expansivity of THF-d– and THF-d–O_2_clathrates, drawing attention to them as an interesting case study for the complex structure and dynamics of this class of material.

## 1. Introduction

**Figure 1:**
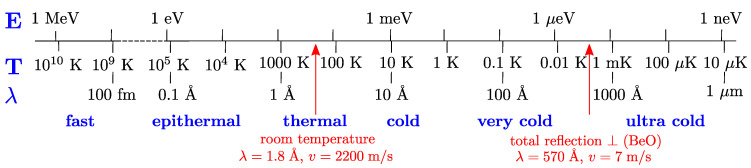
Characterization of different ranges of neutron energies from fast to ultra cold, with conversion to temperature (in thermal equilibrium $E=k_{B}T$) and wavelength according to the de Broglie relation (Equation (1)).

Due to their wide range of dispersion-free low-energy modes, their weak absorption, and Bragg scattering up to atypically high wavelengths, deuterated clathrate hydrates have been identified as a promising class of materials for moderating thermal or cold neutrons (CNs) to very cold neutrons (VCNs) [1,2,3]. The energy *E*, wavelength λ, and velocity *v* of a neutron are related by the de Broglie relation:

(1) $$E=\frac{1}{2}mv^{2}=h\nu =\frac{h^{2}}{2m\lambda^{2}}\;,$$

and are given in Figure 1, where *m* is the neutron mass and *h* is the Planck constant. Neutrons with energies below 5 meV (4 Å) are commonly termed cold neutrons. Very cold neutrons, on the other hand, extend into the long-wavelength tail of typical sources for cold neutrons (the most common cold moderator materials for both spallation and fission neutron sources are liquid deuterium (L$D_{2}$), liquid hydrogen (L$H_{2}$), and liquid or solid hydrocarbons (e.g., methane ${\mathrm{CH}}_{4}$)), with energies below 1 meV (9 Å) down to a few hundreds of neV (greater than several 100 Å), which is the domain of ultra cold neutrons (UCN); see Figure 1.

The scientific potential of novel VCN sources is extensively discussed in [4,5,6,7]. The neutron moderation capabilities of clathrate hydrates stem from their properties as inclusion compounds. By hosting suitable guest molecules, these hydrates can facilitate neutron down-scattering not only through nuclear scattering but also via magnetic scattering.

Nuclear scattering differs in its occurrence within the host lattice compared to the guest molecule. The scattering within the host lattice is primarily dominated by phonon-scattering, whereas guest molecules (such as methane CH_4_ or tetrahydrofuran C_4_H_8_O) introduce localized excitations from rotational, librational, or Einstein modes. These modes, unlike phonons, are not restricted by dispersion relations [2,3]. Paramagnetic guest molecules give rise to magnetic scattering. Notably, dioxygen (O_2_) is of particular interest due to its magnetic triplet ground state with a zero-field splitting of approximately 0.4 meV, corresponding to 4.6 K on a temperature scale (see Figure 1). In a dioxygen-containing clathrate cooled to ≈4.6 K this low excitation energy enables moderation via a cooling cascade mechanism, as described in [1]. In this context, clathrate hydrates allow for a very dense packing of O_2_ molecules, still avoiding magnetic order, as observed, for example, in the antiferromagnetic crystalline α phase of pure dioxygen [8].

**Figure 2:**
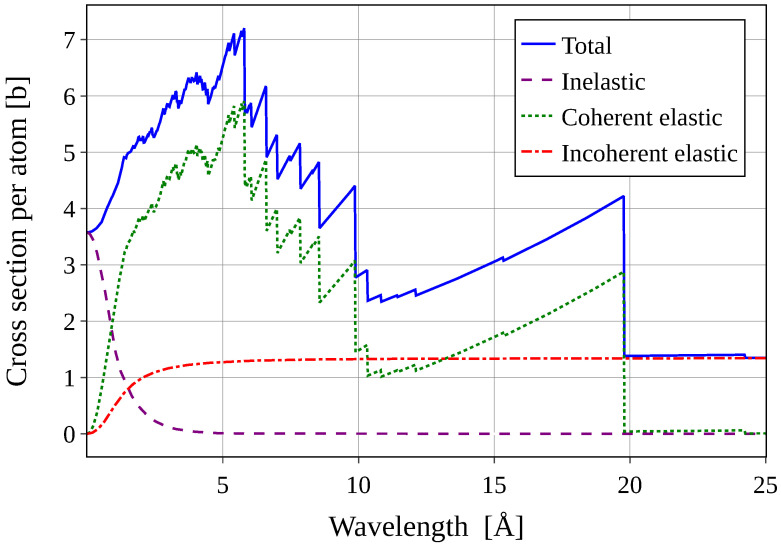
The different contributions to the neutron scattering cross-section of a CS-II hydrate hosting THF-d. The cold neutron range is dominated by coherent elastic Bragg scattering providing excellent diffuse reflection properties for moderator applications (see text). The plot was generated with NCrystal [9] based on simulations described in [10].

Furthermore, the large crystallographic unit cells of clathrate hydrates (see Table 1) give them an additional desirable property for materials employed in a VCN source—coherent elastic scattering in the CN range up to atypically large wavelengths. This can be understood by considering Bragg’s law, which describes the occurrence of coherent elastic scattering in a given material:

(2) $$n\lambda =2d_{hkl}\sin\left(\theta \right)\;,$$

where *n* is the diffraction order, λ is the neutron wavelength, $d_{hkl}$ is the lattice spacing between neighboring hkl-planes, and θ is the diffraction angle. For a given $d_{hkl}$, there exists a maximum λ above which diffraction cannot occur at any angle, referred to as a Bragg edge. The largest-wavelength Bragg edge is known as the Bragg cutoff. Beyond the Bragg cutoff, no coherent elastic scattering takes place (except in forward direction, entering the neutron refractive index). Figure 2 illustrates this phenomenon for a fully deuterated CS-II hydrate structure hosting THF-d.

Ideally, a VCN moderator would feature multiple Bragg edges throughout the CN region and a Bragg cutoff in the low-wavelength VCN region. The added coherent scattering with cross-section $\sigma_{coh}$ increases the path length of diffusive neutron transport in the medium, thus creating more opportunities for down-scattering. For neutrons with wavelengths beyond the Bragg cutoff, the scattering becomes weaker and is dominated by spin incoherent scattering from the deuterons. This allows moderated neutrons to easily escape from the moderator, after which they can be transported to instruments using neutron optical techniques, e.g., neutron guides. In simpler terms, the hydrates serve as a flux trap for cold neutrons through Bragg scattering, covering a range of up to approximately 20 Å for the CS-II structure (the Bragg cutoff of the CS-I structure lays at approximately 17 Å). Once moderated to VCN, these neutrons can be easily extracted.

**Table 1:** Characteristics of the CS-II and CS-I hydrate crystal structure. In the case of THF hydrates, the unit cell formula reduces to $8\;\mathrm{THF}\;\cdot \;136\;H_{2}O$, with the $5^{12}$ cages remaining empty. Table adapted from ([11], p. 60). Note that the lattice parameters are highly dependent on temperature, pressure, and guest molecules. These values are averages at typical conditions of common hydrates.

	CS-II	CS-I
Crystal system	Cubic	Cubic
Space group	Fd$\bar{3}$m (N°227)	Pm$\bar{3}$n (N°223)
Lattice description	Face centered	Primitive
Lattice parameters	a=17.3 Å α=β=γ=90°	a=12 Å α=β=γ=90°
Ideal unit cell formula	$8\left(5^{12}\;6^{4}\right)\;\cdot \;16\left(5^{12}\right)\;\cdot \;136H_{2}O$	$6\left(5^{12}\;6^{2}\right)\;\cdot \;2\left(5^{12}\right)\;\cdot \;46H_{2}O$

The suitability of clathrate hydrates for VCN moderation relies on the following factors:

1. The choice of guest molecules with low absorption and strong scattering;
2. The formation of the hydrate structure with a sufficiently high yield in quantity of the material;
3. A sufficiently high cage occupancy of guest molecules.

Following these criteria, hydrates hosting THF and dioxygen seem particularly promising. While clathrate hydrates are generally non-stoichiometric compounds, THF hydrates are known for reliably forming the CS-II structure from a stoichiometric solution, featuring large crystallographic unit cells and practically full occupation of the $5^{12}\;6^{4}$ cages. On the other hand, dioxygen complicates the manufacturing process but enables effective neutron cooling down to the lowest temperatures through magnetic scattering. While this binary hydrate has been produced before from a slurry by constantly stirring a solution of one part THF in 17 parts of water, pressurizing it with O_2_ and slowly decreasing the temperature [12,13], we present here a different manufacturing method. It involves milling the fully formed THF hydrate in a ball mill, followed by exposing the formed powder to pressurized dioxygen. This method is not only easily scalable for producing large amounts of material, but it is also effective to maximize the yield of CS-II hydrate and the cage occupancy of O_2_.

The application of clathrate hydrates in a moderator requires a reliable method of producing large quantities of the material. Since the moderator has to be cooled to temperatures well below 4 K to be most effective, and the clathrate hydrates have only a low thermal conductivity, it is proposed to immerse a porous block of packed powder in super-fluid helium serving as an effective and neutron non-absorbing medium for cooling. The production of the THF-d clathrate hydrate powder and the addition of the dioxygen can, for practical reasons, not be performed in situ in the moderator tank, so a cold transfer of the material is necessary after its preparation.

In this article, we present the first test of this method along with neutron diffraction data of the obtained samples, confirming the structure and providing an estimate of the cage occupancy. We report the hydrate, or, rather, deuterate (throughout this article, the terms clathrate hydrate or hydrate are used when referring to general structure and properties, while deuterate is used for concrete samples prepared from heavy water), yield in weight percent computed from a multi-phase analysis and an estimate of the occupation of the $5^{12}$ cages. Additionally, we present data on the thermal expansivity of the obtained deuterate.

## 2. Materials and Methods

### 2.1. Manufacturing of THF-d–O_2_ Deuterates

The manufacturing technique for THF-d–O_2_ hydrates presented here follows three basic steps:

1. Forming THF-d hydrates from a stoichiometric solution of THF-d:17·D_2_O.
2. Grinding the THF-d hydrate into a fine powder, providing a large reaction surface.
3. Exposing the powder to a high-pressure O_2_ atmosphere.

The preparation of THF hydrates was previously outlined by the authors [2], (It should be added that the evaporation at room temperature of water (or heavy water) can be neglected compared to that of THF (or THF-d). For THF-d, we observed an evaporation rate of 0.6 mg/min from an open 17 mL vial. The most precise way to achieve an almost exact stoichiometric ratio is adding slightly more THF than necessary and monitoring the evaporation). The materials used in this process are provided in Table 2.

Previous studies have shown the feasibility of creating clathrate hydrates starting from hexagonal ice (Ih) [14,15,16]. However, the grain sizes achieved by grinding ice Ih with a mortar in liquid nitrogen is in the 10 to 100 μm range, with a broad Gaussian distribution [17]. In addition, manual grinding in a mortar is hardly feasible for the amount of material that is needed for moderator applications, which, depending on the size of the moderator, run from 100 g to several kg.

#### 2.1.1. Grinding THF-d Deuterate into Fine Powder

**Figure 3:**
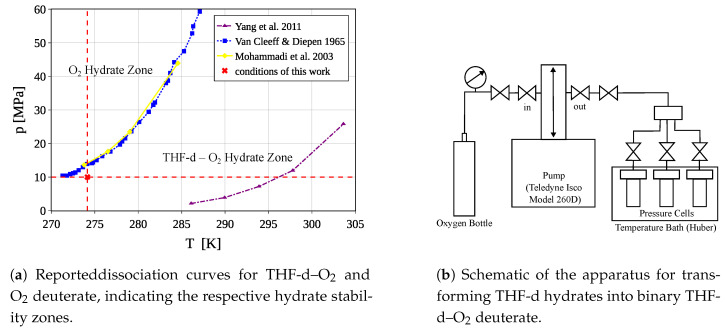
The oxygen bottle is connected via a pressure gauge with the pump system (Teledyne Isco Model 260D, Thousand Oaks, CA, USA), which supplied a constant pressure of 10 MPa, compensating for the O_2_ uptake of the hydrate. The gas was then distributed on three identical pressure cells separated by a valve (Top Industrie, Vaux-le-Pénil, France). The cell’s temperature was kept steadily at 274.15 K with a temperature controller (Huber pilot one, Offenburg, Germany). The conditions for the pressurization were chosen based on the reported literature. See [13,19,20] for the data depicted in Figure 3a.

Milling already-formed THF-d hydrates, rather than grinding ice Ih, as a starting point to form the binary hydrate has multiple advantages. Not only does milling allow us to produce high quantities of fine powders, it also provides a smaller and narrower distributed grain size, suggesting faster and more uniform hydrate formation. Moreover, using THF-d hydrates rather than ice Ih as a starting material changes the necessary thermodynamic conditions for the formation of a binary THF-d–O_2_ hydrate, allowing its formation at lower pressures (see Figure 3a).

An important condition to fulfill in this process, particularly when preparing powders of deuterates, is to maintain low temperatures while preventing the condensation of H_2_O from the laboratory atmosphere. The latter, which could alter both the stoichiometry and the isotopic composition of the sample, can be kept under control by handling the deuterate in a dry nitrogen or argon atmosphere within a glove box or bag.

Preliminary tests indicated that the thermal mass of the 50 mL jar pre-cooled to liquid nitrogen temperature was sufficient to mill for over 4 min at 500 rpm before the jar temperature exceeded 270 K. As milling cycles of 60 s to 100 s yielded similar powders, a shorter time per cycle (90 s) was chosen to avoid compromising the CS-II. The potential introduction of stacking disorders during the grinding and milling of THF-d hydrates was described in [18]. This makes it imperative to validate the structure of these powders.

For the milling, we used a RETSCH PM100 planetary ball mill (Haan, Germany) equipped with a stainless steel grinding jar with a volume of 50 mL and grinding balls with diameters of ⌀=5 mm, ⌀=10 mm, and ⌀=20 mm (this configuration was selected to produce the small quantities required for this work; the same mill supports jars with volumes up to 500 mL). The finest powder was achieved using 10 balls of ⌀=5 mm.

The process was carried out as follows: In a glove bag, the stoichiometric solution THF-d:17·D_2_O was poured into a thin-walled aluminum pan in liquid nitrogen. The solid deuterate forms at the bottom of the aluminum pan within less than a second. It can then be transferred together with the stainless steel balls into the grinding jar, which is under argon and in liquid nitrogen. After the transfer, the jar is closed in the argon atmosphere and placed in the ball mill. To reduce the heat exchange with the environment, the contact surfaces between the jar and the mill were insulated with Teflon and the jar was wrapped in aluminum foil, which also avoided condensation of air humidity on it.

Following milling, the jar was transferred back into the glove bag, reopened under an argon atmosphere, and the cold powder was transferred into a container for neutron scattering analysis of the THF-d deuterate or into the pressure cell for creation of the binary clathrate (Section 2.1.2).

#### 2.1.2. Dioxygen Gassing

To prepare the binary THF-d–O_2_ deuterate, the powder was kept at dry ice temperatures and inserted into a 9.8 mL pressure cell made of stainless steel. The cell was flushed with O_2_, sealed with indium wire, and tightly closed. Subsequently, the cell was maintained at a constant temperature of 274.15 K and a pressure of 10 MPa. This was facilitated by a water bath with a temperature controller and a pump controller that compensated for pressure loss due to O_2_ uptake in the hydrate, ensuring homogeneous filling. A flow chart of this process is depicted in Figure 3b.

Figure 3a shows established hydrate dissociation curves for the O_2_–water system and [13] for the O_2_–THF water system, as reported in [13,19,20]. Temperature and pressure for our preparation were chosen to lie just outside the O_2_–hydrate zone yet securely within the O_2_–THF hydrate zone, optimizing the yield of the binary hydrate. Typically, clathrate formation rates are higher in the initial stages and become slow after several days [15]. After 10 days (this time was mostly defined by instrumental constraints and should be increased for higher cage filling factors) at 274.15 K and 10 MPa, the hydrates were cooled to liquid nitrogen temperatures, the pressure in the cell was slowly released, and the THF-d–O_2_ deuterate was extracted under an argon atmosphere.

### 2.2. Neutron Diffraction at D20

The powder samples of THF-d– and THF-d–O_2_ deuterates were placed under argon atmosphere and liquid nitrogen in cylindrical sample containers made from vanadium, with an inner diameter of 6 mm and a wall thickness of 0.1 mm. The high-intensity two-axis diffractometer D20 at the Institut Laue-Langevin was operated using the (115) reflection of a Germanium (113) monochromator at a wavelength of λ=1.546 Å. At a takeoff angle of 90°, this results in a lattice-spacing resolution of $\Delta d/d=3\times 10^{-3}$. The high flux at D20 allows us to obtain diffraction patterns between double scattering angles of 4° and 150°, with satisfactory statistics in about 30 min. The objective of the experiments at D20 was to validate that (1) the ball milling procedure described above preserves the CS-II structure and (2) the whole sample preparation prevents intake of water from air humidity at non-negligible levels. In a first step, neutron diffraction experiments were performed using a sample of the milled material before gassing. For comparison, the same experiments were also performed for a sample prepared by manual grinding in a mortar instead of milling. Both datasets were then compared with previous data obtained from THF-d deuterate produced by quenching a stoichiometric solution [2]. In a subsequent step, the manually ground and ball-milled samples were pressurized with O_2_ as described above, and the resulting THF-d–O_2_ deuterates were investigated. The ball mill sample was divided into two sub-samples from the top and bottom of the pressure cell, respectively. Comparison of the diffraction data from these samples allowed us to detect a possible difference of O_2_ content due to the different location of the powder with respect to the oxygen inlet valve.

To evaluate the thermal expansion, as described in Section 3.3, the two binary samples were measured at temperatures ranging from 1.5 K to 80 K, and the simple THF-d deuterate from 2 K to 100 K, in increments of 5 K.

### 2.3. Rietveld Refinement

The raw data were analyzed to derive thermal expansion, and structure refinements were performed in Fullprof [21,22]. The Rietveld method [23], a crystallographic least-squares refinement method, was employed to validate the structure. This method provides information on lattice constants and positional and thermal parameters, as well as guest occupancies for every crystalline phase within the sample. It is important to note that instrumental and background parameters are established concurrently with the parameters of the structural model. As a result, the estimated standard deviations derived from such a method partly reflect instrumental and sample imperfections but not necessarily other systematic errors.

The THF-d molecule was treated as a rigid body, with its position and orientation within the large cage as free-fitting parameters. The O_2_ molecule was treated as a free atom at the corresponding site. In addition to the phase under investigation (THF-d deuterates and binary THF-d–O_2_ deuterates), the refinement included the aluminum of the sample environment and residual hexagonal ice. Phase fractions are determined through a scale factor as part of a multi-pattern refinement procedure (see, e.g., [24]). This allows quantitative analysis of the mass percentage of each phase in the sample. The weight percentage $W_{j}$ for the phase *j* can be calculated as in [25]:

(3) $$W_{j}=\frac{S_{j}Z_{j}M_{j}V_{cj}}{\sum_{i}^{N}S_{i}Z_{i}M_{i}V_{ci}}\;,$$

with *S*, *Z*, *M*, and *V* being the scale factor, the number of formula units per unit cell, the mass of one formula unit, and the unit-cell volume, for each phase *j* and *i*, respectively. Fullprof also accounts for the multiplicities of each site, for occupation numbers unequal to 1, via the factor $f_{j}$, and the micro-absorption of neutrons (Brindley factor $t_{j}$) of each phase (see Ref. [26] for details):

(4) $$\begin{array}{cc} W_{j}=\frac{S_{j}Z_{j}{f_{j}}^{2}M_{j}V_{j}/t_{j}}{\sum_{i}^{N}S_{i}Z_{i}{f_{i}}^{2}M_{i}V_{i}/t_{i}} & =\frac{S_{j}\;\cdot \;\mathrm{ATZ}\;\cdot \;V_{j}}{\sum_{i}^{N}S_{i}\;\cdot \;{\mathrm{ATZ}}_{i}\;\cdot \;V_{i}}\;,\\ \mathrm{with}\;\;\mathrm{ATZ} & =Z_{j}{f_{j}}^{2}M_{j}/t_{j}. \end{array}$$

As the two phases within the refinement have very similar absorption, $t_{j}$ is ∼1. In a stochiometric compound, $f_{j}$ reduces to 1, as the multiplicities are calculated by dividing the Wyckoff multiplicity *m* of a particular site by the general multiplicity *M*. This is the case for the THF-d deuterate, where Equation (4) reduces to Equation (3). In the case of non-stochiometric compounds, as in the binary THF-d–O_2_ deuterate, these multiplicities are calculated by

(5) $$f=\frac{Occ\;M}{m}\;,$$

where Occ is the occupation number used in the refinement. This allows us to compute the cage occupancy of the O_2_ in the small cages, by allowing the occupation number Occ of the respective O_2_ to be a free refinement parameter and using Equation (4) to calculate the weight percentage.

### 2.4. Evaluation of the Thermal Expansivity

The thermal expansivity is isotropic for a cubic crystalline structure and is defined as the partial derivative of the lattice parameter *a* under constant pressure *p*: [27,28]

(6) $$\alpha =\frac{1}{a}{\left(\frac{\partial a}{\partial T}\right)}_{p}\;.$$

The expansion of the cell parameter at a reference temperature $T_{0}$, $a_{0}=a\left(T_{0}\right)$, to a temperature *T* can then be expressed as

(7) $$a\left(T\right)=a_{0}\left(1+\alpha \Delta T\right)\;,$$

where $\Delta T=T-T_{0}$. Generally, α is a function of temperature. Since the *T*-dependency of α is small, it can be expanded around some $\alpha_{0}=\alpha \left(T_{0}\right)$ and approximated by [29,30,31]

(8) $$\alpha \left(T-T_{0}\right)\approx \alpha_{0}+\alpha_{1}\left(T-T_{0}\right)+\alpha_{2}{\left(T-T_{0}\right)}^{2}\;.$$

This quantity can be determined from the raw data independently of any refinement. Given a high-quality diffractogram, a peak-finding algorithm (see, e.g., [32]) can identify a number of *N* peaks. The positions of these peaks in 2θ are then monitored as a function of temperature. Since the determination of the peak maximum is limited by the angular resolution of the instrument (this was 0.5 deg for the configuration in which the reported data was obtained), this process can be improved by fitting each of the peaks with a Lorentzian peak shape function over its FWHM and computing the peak center μ and its standard deviation σ. Using Bragg’s law (Equation (2)), the lattice spacing can then be expressed by

(9) $$d_{hkl}=\frac{\lambda }{2\sin\left(\theta \right)}$$

The parameters μ in *d*-spacing can then be plotted with their respective σ as a function of *T*. The thermal expansivity α(T) can then be obtained using Equation (7) by computing the relative expansion of the lattice spacing from a reference temperature $T_{0}$

(10) $$\alpha \left(T\right)=\frac{\Delta d}{d\Delta T}=\frac{d\left(T\right)-d\left(T_{0}\right)}{d\left(T_{0}\right)\left(T-T_{0}\right)}\;,$$

for all measured *T* and finding the coefficients $\alpha_{0}$, $\alpha_{1}$, and $\alpha_{2}$ from Equation (8) that fit the data.

## 3. Results

### 3.1. Fine Powders of THF-d Deuterates

**Figure 4:**
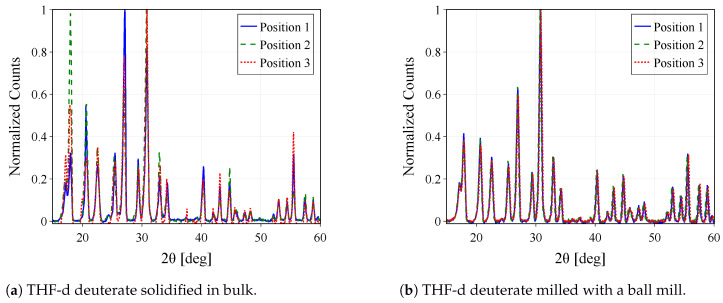
Diffraction patterns measured for three different angular positions of the sample in the beam ($0^{{}^{\circ}},40^{{}^{\circ}},90^{{}^{\circ}}$) for three different samples of THF-d deuterate in a limited range of $2\theta =[15^{{}^{\circ}},60^{{}^{\circ}}]$, at a temperature of 2 K. While the bulk sample (**a**) shows clear texture (manifesting as a change of the intensity profile as a function of the sample rotation), the three datasets from the milled sample (**b**) match almost perfectly.

Prior to investigating the binary hydrate samples, we confirmed through neutron diffraction that the CS-II structure consistently forms as a polycrystalline solid with minimal residual ice. This was observed regardless of whether the solution was quenched in liquid nitrogen or slowly cooled in a cryostat, as presented in [2]. However, the diffraction data also revealed clear texture effects. Figure 4 presents diffractograms of two distinct samples of THF-d deuterates: solidified in bulk (a), and milled with a ball mill (b). A rotation of the sample allows an examination of the influence of texture on the diffraction pattern. Notably, rotations by $40^{{}^{\circ}}$ and $90^{{}^{\circ}}$ exhibit a clear effect in the intensity profile of the bulk sample, while minimal effects are observed in both the milled and ground samples. This demonstrates the successful mitigation of texture through grinding and milling, with the structure remaining fully intact. This conclusion is supported by the consistent peak positions in the diffractograms, as verified through the Rietveld method.

### 3.2. Structure Analysis of the Binary THF-d–O_2_ Deuterate

**Figure 5:**
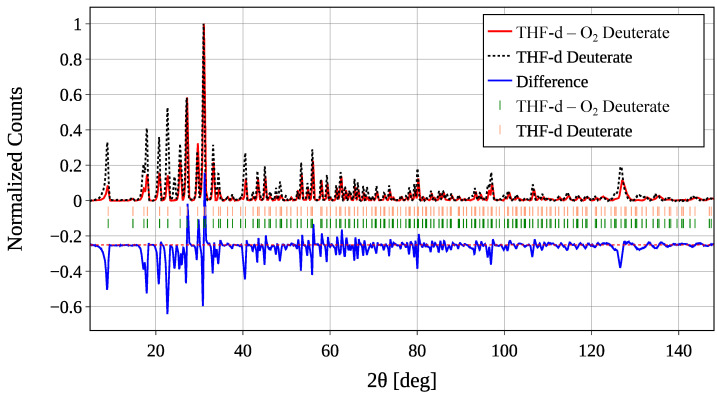
Comparison of the diffraction patterns of a powder of THF-d deuterate (black dotted line, peak position indicated in light salmon) and the bottom sample of THF-d–O_2_ deuterate (red line, peak position indicated in green) at a temperature of 2 K. The difference of the two diffractograms is given in blue and shifted along the vertical axis by −0.25, indicated by a red dashed line for better visibility. See text for details.

A comparison of the THF-d and THF-d–O_2_ samples is shown in Figure 5. The two samples show very similar peak positions while exhibiting different intensity profiles. This provides clear evidence that the cubic *Fd3m* structure typical for CS-II hydrates is maintained throughout the manufacturing process. Furthermore, the observed intensity differences indicate a modification of the *structure factor* due to the introduction of O_2_ into the $5^{12}$ cages.

**Figure 6:**
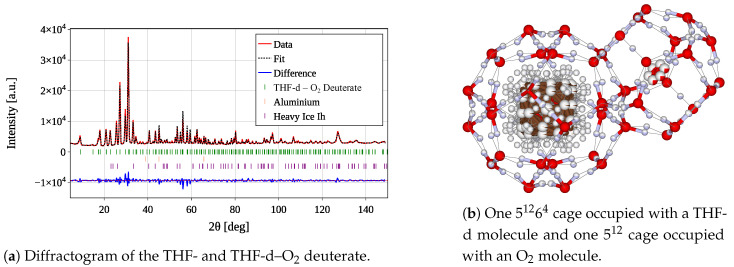
Diffractogram of the bottom sample of the THF-d and THF-d–O_2_ deuterate (**a**), one representative $5^{12}6^{4}$ cage occupied with a THF-d molecule and one $5^{12}$ cage with O_2_ computed from the refined pattern (**b**). The original data are depicted in red. The fit from the refinement (dashed black) was obtained through a multi-phase Rietveld refinement. The residuals are depicted in blue and shifted by $-9.4\times 10^{-5}$ along the vertical axis, indicated by a red dashed line. The peak positions for each considered phase are indicated by vertical lines. The reliability factors for a refinement over the given 2θ-range are computed to be $R_{p}=18.0$, $R_{wp}=20.7$, and $R_{e}=2.85$.

A complete Rietveld refinement of the THF-d–O_2_ deuterate, as depicted in Figure 6a, confirms the CS-II structure. At 2 K, the determined lattice parameter is a=17.12202±0.00031Å, which agrees reasonably well with the reported data in [12] (see also Section 3.3).

The reliability factors of the refinement [33] at a base temperature of 2 K for a refinement spanning the entire 2θ-range (4.5–148.5°) are computed as $R_{p}=18.0$, $R_{wp}=20.7$, $R_{e}=2.85$. These factors can be further reduced to $R_{p}=12.5$, $R_{wp}=14.0$, $R_{e}=2.21$ by limiting the 2θ-range to 4.5–60.5°. Both the top and bottom samples (see Section 2.1.2) exhibit very similar purity of the CS-II hydrate phase, with weight percentages of 99.41±1.67% for the top sample and 98.78±2.02% for the bottom sample. The primary impurity is assumed to be residual ice Ih, with weight percentages of 0.59±0.27% and 1.22±0.20%. Similar results were obtained for temperatures ranging from 2 K to 80 K.

One of the primary objectives of this study was to estimate the O_2_ occupation within the $5^{12}$ cages. In this context, it is assumed that the larger $5^{12}6^{4}$ cages are exclusively and fully occupied by the significantly larger THF-d molecule (see Figure 6b). The longest end-to-end distance within the THF-d and oxygen molecules, calculated from the molecular coordinates provided in [34,35], is 4.16 Å and 1.23 Å, respectively. Although the occupation of the $5^{12}6^{4}$ cages is not entirely excluded, it is considered negligibly small based on the stoichiometric composition of the THF-d deuterate (see also [12]). The occupation of $5^{12}$ cages was determined by treating the occupation number occ of the oxygen within the cage as a free-fitting parameter. Given that its site has a Wyckoff multiplicity of m=32, and the general multiplicity of the *Fd3m* space group is M=192, the cage occupation of O_2_ can be calculated using Equation (5).

The refinements of the data of the top and bottom sample at 2 K gave very comparable occupation numbers of 0.798±0.015 and 0.801±0.011, respectively, indicating a homogeneous formation of the deuterate throughout the pressure cell. As a counter check, we can compare the computed volume density of the hydrate with that of a fully occupied THF-d and THF-d–O_2_ deuterate, which is $1.29g/{\mathrm{cm}}^{3}$ for the evaluated cell parameter. The volume density of the refined structure can be calculated by adding up all the mass contributions of the unit cell divided by its volume:

(11) $$\rho =\sum_{j}^{N}\frac{f_{j}Mj}{a^{3}}\;,$$

which results in $1.257g/{\mathrm{cm}}^{3}$ and $1.258g/{\mathrm{cm}}^{3}$ for the top and bottom sample, respectively. The difference from the fully occupied deuterate of about $0.03g/{\mathrm{cm}}^{3}$ accounts for 20% of the $5^{12}$ cages being empty.

### 3.3. Thermal Expansivity

**Figure 7:**
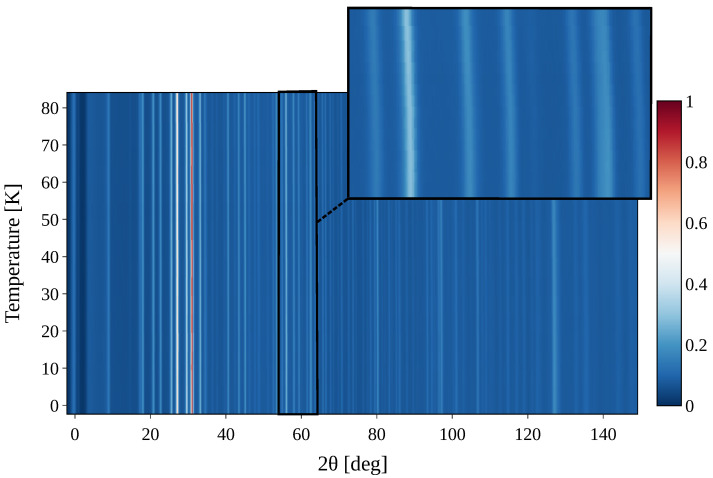
Heatmap of the diffractograms measured at temperatures from 1.5 K to 80 K. The color bar indicates normalized counts. The range $2\theta =[54^{{}^{\circ}},64^{{}^{\circ}}]$ is magnified in the upper right corner.

Figure 7 shows the diffractograms of the THF-d–O_2_ deuterate bottom sample during cool-down arranged as a heatmap. The color bar indicates the normalized counts, while *T* is plotted on the vertical axis. Magnifying the range from $2\theta =[54^{{}^{\circ}},64^{{}^{\circ}}]$ clearly shows how the lines deviate from the vertical due to thermal expansivity of the sample.

The parameters of the peak finding algorithm, described in Section 2.4, were carefully selected to prioritize those peaks in the analysis, with the highest prominence, i.e., N=19 peaks, for each given temperature for the THF-d–O_2_ deuterate and N=15 for the THF-d deuterate. Recalling that each peak corresponds to a specific lattice plane distance $d_{hkl}$, the temperature dependence of this lattice spacing can be calculated using Equation (10). Calculating Δd/d for each diffractogram and computing the average yields the following:

(12) $$\bar{\alpha }\left(T\right)=\bar{\frac{\Delta d}{d\Delta T}}=\frac{1}{N}\sum_{i=0}^{N}\frac{d_{i}\left(T\right)-d_{i}\left(T_{0}\right)}{d_{i}\left(T_{0}\right)\left(T-T_{0}\right)}\;.$$

**Figure 8:**
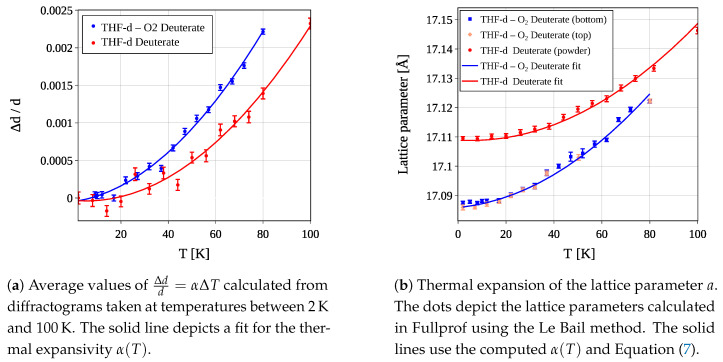
Thermal expansivity α(T) calculated as outlined in Equation (10) (**a**) and expansion of the lattice parameter of THF-d–O_2_ deuterate and THF−d deuterate (**b**). The thermal expansion of the lattice parameter compares the values obtained with the Le Bail method (blue, red, and pink circles) with the prediction resulting from $a\left(T\right)=a_{0}\;\alpha \left(T\right)$ (solid lines), where $a_{0}=a\left(T_{0}=2K\right)=\left(17.0867\pm 3.2\times 10^{-4}\right)Å$ for the the THF-d–O_2_ deuterate and $(17.1094\pm 4.1\times 10^{-4})Å$ for the THF−d deuterate, respectively.

Figure 8a presents the computed values of $\frac{\Delta d}{d}$ across all measured temperatures $T_{j}$ for both the THF-d–O_2_ and the THF−d deuterate. The solid lines represent a fit using Equation (8). The fit parameters are given in Table 3. To estimate the uncertainty of the fit parameters, a Monte Carlo method was employed. Generating datasets with random variations within the specified uncertainty of the original data and performing fits on these datasets allows us to compute the average fit parameters and their corresponding uncertainties.

In the subsequent step, the calculated thermal expansivity was employed for cross-checking the thermal expansion derived from the refinement. The lattice parameter refinement was conducted using the Le Bail method [26,36]. This method focuses on refining the lattice parameter for a predetermined space group rather than performing a full refinement. The reliability factors obtained were $R_{p}=6.74$, $R_{wp}=8.13$, and $R_{e}=2.39$ for the THF-d–O_2_ deuterate, and $R_{p}=7.01$, $R_{wp}=8.50$, and $R_{e}=2.85$ for the THF−d deuterate at T=2K. Similar results were observed at other temperatures.

Figure 8b illustrates the expansion of the lattice parameter *a* as a function of temperature. The circles represent computed values of *a* using the Le Bail method. The solid lines depict a(T) as in Equation (7), utilizing the α(T) values from Figure 8a, and $a_{0}$ denotes the lattice parameter at the base temperature of T=2K, which is $17.0867\pm 3.2\times 10^{-4}\;Å$ for the THF-d–O_2_ deuterate and $17.1094\pm 4.1\times 10^{-4}\;Å$ for the THF−d deuterate, respectively.

## 4. Discussion

**Figure 9:**
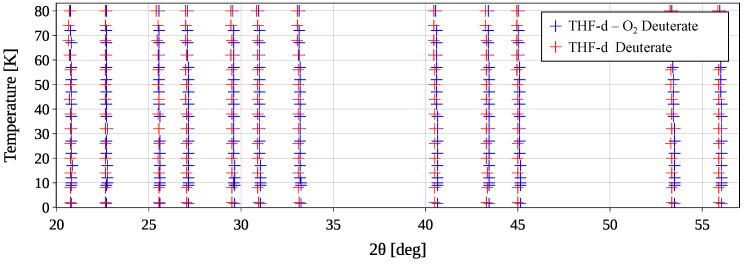
Selected peak positions in a 2θ range between $20^{{}^{\circ}}$ and $57^{{}^{\circ}}$ for the measured temperature range. The peak positions of the THF−d deuterate are consistently shifted towards lower angles compared to the THF-d–O_2_ deuterate, indicating a larger unit cell.

The apparent reduction in unit cell size for the binary THF-d–O_2_ deuterate, compared to THF−d deuterate, might raise concerns about the reliability of the Rietveld refinement results. However, this outcome is not only supported by the raw data but is also consistent with the existing literature. Figure 9 displays the peak positions in the specified temperature range between $2\theta =20^{{}^{\circ}}$ and $2\theta =57^{{}^{\circ}}$. The peak positions of THF−d deuterate are visibly shifted towards lower angles, indicating a larger unit cell. In fact, the ratio of two identical hkl-plane distances $d_{hkl}$, calculated from the first peak position in Figure 9 using Equation (9), corresponds to the ratio of their respective lattice parameters determined by the Le Bail method. For the samples at T=2K, this is expressed as follows:

(13) $$\frac{d_{O_{2}}}{d_{\mathrm{no}\;O_{2}}}=\frac{\sin\left(\theta_{\mathrm{no}\;O_{2}}\right)}{\sin\left(\theta_{O_{2}}\right)}=99.84\pm 0.008\%\;.$$

In comparison, the ratio of the lattice parameters calculated with the Le Bail method at the same temperature is

(14) $$\frac{a_{O_{2}}\left(T=2K\right)}{a_{\mathrm{no}\;O_{2}}\left(T=2K\right)}=\frac{17.087(5)}{17.109(4)}=99.871\pm 0.004\%\;.$$

It is worth noting, though, that the value obtained for the lattice parameter of the binary deuterate a(T=80 K)=17.122(1)Å is slightly larger than the one reported for the corresponding hydrate in [12] (a(T=100 K)=17.1143(5)Å). This difference could be attributed to both deuteration and the filling of the $5^{12}$ cages. The contribution of the cage filling, which is not reported in [12], can significantly impact the unit cell expansion, particularly at higher temperatures [16]. Deuteration generally weakens H-bonds in ice Ih and clathrate hydrates, resulting in longer bond distances [16], which could contribute to the observed discrepancy. The values of the lattice parameter of the THF-d deuterate seem to be in reasonable agreement with the literature [29,31,37], which shows variations of up to 0.03Å at comparable temperatures.

The observed shrinking of the unit cell upon the introduction of dioxygen into the $5^{12}$ cages could be attributed to guest–host attraction at low temperatures. Molecular dynamics simulations indicate an expansion of the H_2_O network in the CS-I structure when the cages are left empty [38,39]. This explanation finds support in data on N_2_- and Ne-hydrates reported by [40], which shows a lattice shrinkage compared to the empty CS-II structure, and a larger lattice parameter for the significantly smaller Ne atom.

The analysis of thermal expansion proved to be more complex than anticipated. Earlier published data show a large variety in the lattice parameter [29,31,37] and its thermal expansion [29,31,41]. This variation could be due to differences in cage filling and preparation methods, isotopic effects, or experimental uncertainties, as described in [16]. The strong non-linearity at low temperatures is characteristic of the composition of clathrate hydrates as Einstein solids [42,43]. The low frequencies of the guest molecule’s rattling modes allow for excitations at very low temperatures. This is exactly the property that we intend to exploit for neutron moderation. It is also visible in the thermal expansivity of the lattice parameter, which is heavily influenced by the coupling of these modes with the lattice structure [44].

The low onset temperature of this Einstein mode explains the particular form of α(T) observed in Figure 8a. It also accounts for the steeper increase in the thermal expansivity of THF-d–O_2_ deuterate compared to THF−d deuterate. The higher fraction of O_2_, nearly twice as much, allows a wider range of rattling excitations, resulting in increased expansivity at low temperatures. This effect vanishes at higher temperatures when the excitation spectrum is dominated by acoustic modes of the host lattice.

Further exploration of this effect would require additional data at higher temperatures and a more sophisticated expansion model, as complex interplay between guest–host interactions and lattice dynamics makes the study of thermal expansion in clathrate hydrates an intricate subject. Elements of such a model have been introduced by the authors of [16].

## 5. Conclusions

In this article, we presented an effective and reliable technique for the preparation of binary clathrate hydrates hosting THF and dioxygen, emphasizing their potential applications in neutron moderation. The method starts from forming THF hydrates from a stoichiometric solution, followed by milling and pressurization. This approach is expected to be scalable for producing large quantities of high-purity material. The THF-d–O_2_ deuterate studied in this work exhibited abundances up to 99.41±1.67%. The $5^{12}$ cage fillings with dioxygen of 80.1±1.1% could likely be improved by adjusting the pressurization conditions.

Additionally, we report previously unavailable data on the thermal expansivity of the binary THF-d–O_2_ deuterate. This dataset could be complemented with studies on other binary hydrates hosting molecules with different attraction and repulsion properties in the $5^{12}$-cage. Such studies would provide valuable data for hydrate prediction models and enhance our understanding of hydrate thermal expansivity by modeling them as Einstein solids. 

## Figures and Tables

**Table 2:** The materials used for the manufacturing of binary clathrate discussed in this work.

Component	Supplier	Purity
Dioxygen (O_2_)	Air liquide, Paris, France	≥99.5%
THF (C_4_H_8_O)	Eurisotop, Gif-sur-Yvette, France	≥99.9%
THF-d (C_4_D_8_O)	Eurisotop	isotopic purity ≥ 99.5%
Heavy water (D_2_O)	Eurisotop	isotopic purity ≥ 99.9%

**Table 3:** Fit parameters for the thermal expansivity α(T) described in Equation (8) for the two investigated samples.

	THF-d–O_2_ Deuterate	THF-d Deuterate
$\alpha_{0}$	$-2.56\times 10^{-5}\pm 9.9\times 10^{-6}$	$-3.17\times 10^{-5}\pm 2.5\times 10^{-5}$
$\alpha_{1}$	$4.00\times 10^{-6}\pm 6.5\times 10^{-7}$	$-2.34\times 10^{-6}\pm 1.3\times 10^{-6}$
$\alpha_{2}$	$3.06\times 10^{-7}\pm 8.3\times 10^{-9}$	$2.49\times 10^{-7}\pm 1.3\times 10^{-8}$

## Data Availability

The diffraction data that support the findings of this article are available under http://doi.org/10.5291/ILL-DATA.1-10-57 and http://doi.org/10.5291/ILL-DATA.EASY-1218 reference number 1-10-57 and EASY-1218.

## Abbreviations

The following abbreviations are used in this manuscript:

UCN	Ultra cold neutron
VCN	Very cold neutron
MC	Monte Carlo
THF	Tetrahydrofuran
THF-d	Deuterated tetrahydrofuran
